# Do climate envelope models transfer? A manipulative test using dung beetle introductions

**DOI:** 10.1098/rspb.2008.1801

**Published:** 2009-02-25

**Authors:** Richard P. Duncan, Phillip Cassey, Tim M. Blackburn

**Affiliations:** 1 Bioprotection Research Centre, Lincoln UniversityPO Box 84, Lincoln 7647, New Zealand; 2 Landcare ResearchPO Box 40, Lincoln 7640, New Zealand; 3 Centre for Ornithology, School of Biosciences, University of BirminghamEdgbaston, Birmingham B15 2TT, UK; 4 Institute of Zoology, ZSLRegents Park, London NW1 4RY, UK

**Keywords:** species distribution models, bioclimatic envelope, dispersal limitation, range limits

## Abstract

Climate envelope models (CEMs) are widely used to forecast future shifts in species ranges under climate change, but these models are rarely validated against independent data, and their fundamental assumption that climate limits species distributions is rarely tested. Here, we use the data on the introduction of five South African dung beetle species to Australia to test whether CEMs developed in the native range can predict distribution in the introduced range, where the confounding effects of dispersal limitation, resource limitation and the impact of natural enemies have been removed, leaving climate as the dominant constraint. For two of the five species, models developed in the native range predict distribution in the introduced range about as well as models developed in the introduced range where we know climate limits distribution. For the remaining three species, models developed in the native range perform poorly, implying that non-climatic factors limit the native distribution of these species and need to be accounted for in species distribution models. Quantifying relevant non-climatic factors and their likely interactions with climatic variables for forecasting range shifts under climate change remains a challenging task.

## 1. Introduction

Climatic constraints are a potentially key factor limiting species' distributions (Gaston 2003). Support for this view includes the concordance between species current range limits and isoclines of climate parameters (particularly temperature; Root 1988; Newton & Dale 1996), and the palaeoecological record documenting shifts in species distributions in response to past climatic changes (Coope 1995; Graham *et al*. 1996). There is now unequivocal evidence that the Earth's climate is warming (Jones & Mann 2004), and the forecasted changes in climate should therefore significantly alter species' ranges. Indeed, there is increasing evidence that species are already responding to recent climate changes by adjusting their geographic and altitudinal distributions (Crick *et al*. 1997; Pounds *et al*. 1999; Thomas & Lennon 1999; Parmesan & Yohe 2003; Perry *et al*. 2005; Parmesan 2006), leading, in some cases, to population or species extinction (Pounds *et al*. 1999; McLaughlin *et al*. 2002). Predicting the impacts of climate change on the likely future distributions of species is of fundamental importance in anticipating and planning for conservation needs.

Many studies have tried to quantify how species' ranges will alter under different climate change scenarios (Walther *et al*. 2002; Parmesan & Yohe 2003). A standard approach is to construct a climate envelope model (CEM) quantifying the relationship between a species' known distribution and climatic variables. This model is then used to forecast shifts in the species distribution under future climates (Guisan & Thuiller 2005; Botkin *et al*. 2007). Despite the fact that this approach underpins most attempts to model climate change impacts on species distributions, the method is subject to numerous sources of uncertainty (Hulme 2003; Araújo & Guisan 2006; Barry & Elith 2006; Heikkinen *et al*. 2006; Beale *et al*. 2008), there have been few attempts to validate the models independently.

A fundamental source of uncertainty is the assumption that climate limits species distributions, an assumption that is rarely tested (Parmesan *et al*. 2000). However, we know that factors other than climate can, and do, prevent species from occupying areas that would otherwise be climatically suitable (Gaston 2003). These factors include biotic interactions such as competition, predation and parasitism, dispersal limitations created by geographic barriers, and environmental constraints such as soil or geological conditions. Indeed, recent studies have shown that including spatial variation in biotic interactions, in addition to climate variables, can improve the fit of species distribution models (Heikkinen *et al*. 2006; Araújo & Luoto 2007).

The extent to which climate (versus other factors) limits species distributions, and hence the validity of attempts to model distributional changes using CEMs, is difficult to assess given the large scale over which these constraints operate. One approach has been to try to validate CEMs using independent data, typically from the past distributions of species under different climatic conditions or from the current distributions that arise from introducing species to new regions. For example, if CEMs based on a species' current native distribution can accurately predict its past distribution under different climates (Hill *et al*. 1999; Araújo *et al*. 2005), or its distribution in a new region to which it was introduced by humans (Beerling *et al*. 1995; Thuiller *et al*. 2005; Mau-Crimmins *et al*. 2006), the implication is that climate limits distribution because the distribution tracks climate variation over time or space.

However, even when attempts are made to validate the CEMs independently, additional factors invariably cloud interpretation. First, CEMs implicitly assume that species are at equilibrium with their climatic conditions (Hulme 2003; Guisan & Thuiller 2005). Dispersal limitations may mean that current distributions have not reached equilibrium (Svening & Skov 2004) or result in past distributional shifts lagging behind climate changes (Green *et al*. 2008). Similarly, because they are slow to spread, introduced species may not yet have reached their potential distribution in an invaded range (Peterson 2003). Mismatches between climate distributions in the native range and those predicted for introduced ranges have further been attributed to niche shifts, possibly associated with the rapid evolution of species when introduced to novel environments, which may allow them to advance beyond the limits of their climate distribution in the native range (Broennimann *et al*. 2007; Fitzpatrick *et al*. 2007; Urban *et al*. 2007). Second, CEMs are subject to other sources of error related to the type and quality of data available, and specification of the models relating distribution to climate (Barry & Elith 2006). In most cases, it appears very difficult to separate sources of uncertainty related to data quality and model fitting from the more fundamental question of whether climate actually limits species distributions.

Our aim here is to test the validity of CEMs using data from a large-scale manipulation that overcomes some of these difficulties. Between 1969 and 1984, 4786 releases of 52 dung beetle species were conducted across Australia as part of a programme implemented by Australia's Commonwealth Scientific and Industrial Research Organisation. The aim was to establish dung beetle populations in cattle farming areas in order to deal with the large quantities of cattle dung that accumulated in pastureland, and the associated nuisance fly problems (Bornemissza 1976). Although Australia has a highly diverse native dung beetle fauna (more than 480 species; Matthews 1972, 1974, 1976) many of them are endemic, appear to be adapted to non-agricultural ecosystems and specialize on marsupial dung (Doube *et al*. 1991; Hill 1996; Vernes *et al*. 2005).

**Figure 1:**
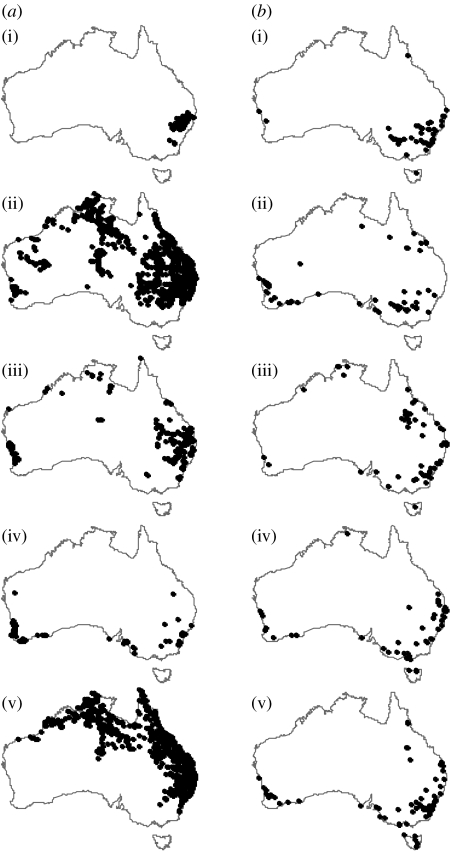
Distribution of release locations across Australia for five species of dung beetle, each released at more than 100 locations. Release locations where the populations (*a*) persisted and (*b*) failed to persist. (i) *Euoniticellus africanus*, (ii) *Euoniticellus intermedius*, (iii) *Onitis alexis*, (iv) *Onthophagus binodis* and (v) *Onthophagus gazella*.

The Australian dung beetle introduction programme has several features that uniquely lend it to testing the validity of the CEM approach. First, many of the dung beetle species widely introduced to Australia were native to South Africa, which has a latitudinal range that overlaps that of Australia, with species selected for release in Australia on the basis of their climatic compatibility with that region (Bornemissza 1976). Dung beetles were subsequently released at numerous sites spanning a wide range of climatic conditions across Australia (figure 1). Second, dung beetles were transferred to sites and released in a controlled manner, and the outcome of those releases (whether the species persisted at that site or not) was extensively monitored. Because the releases were part of a coordinated programme, the number of individuals released per site was large (median=500 individuals per release) and substantially less variable than in invasion studies where the numbers of individuals escaping or released is beyond the study's control. Consequently, for most dung beetle species, variation in the number of individuals released was not significantly related to whether they persisted or not (R. P. Duncan, P. Cassey & T. M. Blackburn 2008, unpublished data), unlike other studies where substantial variation in introduction effort typically leads to this being a primary determinant of success (Lockwood *et al*. 2005; Colautti *et al*. 2006; Hayes & Barry 2008). This ensured that the absence of a species at a release location was not due to dispersal limitation. Third, dung beetle species introduced to Australia were raised in laboratory populations prior to release into the wild, where they were screened for parasites and diseases. Species released in Australia were therefore free of these natural enemies. Dung beetles in Australia are probably predated by birds, lizards, marsupials and introduced foxes and cane toads, but predation pressure is thought to be lower than in Africa and not considered a ‘serious biological threat’ (Bornemissza 1976). Finally, owing to the nature of the introduction programme, dung beetles were released at sites where their primary resource, dung, was highly abundant. This means that failure to establish at a site was not a consequence of resource limitation.

Dung beetles were therefore introduced throughout a new region having climate similar to their native range, but in which other key constraints, notably dispersal limitation, natural enemies and resource limitation, were absent. Under these circumstances, we would expect climate to be a major factor limiting dung beetle ranges, which we test by constructing CEMs based on dung beetle distributions in Australia. The ability of these models to predict dung beetle distributions in Australia sets the standard for how we expect models to perform when climate actually does limit distribution. We then compare how these models perform with those in which Australian distributions are predicted from CEMs derived in native South Africa. If the assumptions underlying the CEM approach hold then the models should be transferable: a CEM derived in the native range, where we assume climate limits distribution, should predict distribution in Australia about as well as a CEM derived in Australia, where we strongly expect climate to be the limiting factor.

## 2. Material and methods

### (a) Dung beetle releases in Australia

Our primary data source was the record of original dung beetle releases in Australia between 1969 and 1984 (Tyndale-Biscoe 1996). From the 4786 original releases of dung beetle species, we extracted the 3446 records for which the location (latitude and longitude) and the outcome of the release (whether the population persisted or not) were recorded. Most sites where beetles were released were revisited on one or more occasions to determine the outcome, with the average time between release and the last visit to a site being just over 4 years. If a species was found at the release site during the last visit, we scored the release as successful (the population persisted); if absent, we scored the species as having failed to persist.

The 3446 releases included data for six species each released at 100 or more sites. One of these species (*Hister nomas*, in the family Histeridae) is not a true dung beetle (family Scarabaeidae) and we lacked data on its native distribution in South Africa (see below). We therefore limited our analyses to the five species of true dung beetle recorded as being released in Australia at more than 100 sites for which we had data on the location and outcome of the Australian releases, along with the data on the distribution of the species in its native range. The five species were the following: *Euoniticellus africanus*; *Euoniticellus intermedius*; *Onitis alexis*; *Onthophagus binodis*; and *Onthophagus gazella*. The numbers of successful and failed releases for each species in Australia are shown in table 1, with the distribution of release sites shown in figure 1.

### (b) Species occurrence in the native range

The five species each released at more than 100 sites in Australia are all native to South Africa, although the ranges of three species extend further north. The distribution of Scarabaeidae in South Africa has been particularly well documented, and we obtained presence data on the distribution of dung beetle species in this region from the database described in Koch *et al*. (2000). This database contained 8399 distribution records referenced by latitude and longitude, for 482 species. Of these, 8099 records were from South Africa. Given that the distribution of dung beetles was particularly well documented in South Africa, we used the data on the distribution of the species in this country to construct CEMs quantifying native range–climate relationships. For the five species of interest, we supplemented the data in Koch *et al*. (2000) with distribution data from two additional sources: (i) Bornemissza (1976), which records the locations where dung beetles were collected in South Africa for transport to and release in Australia and (ii) locations in South Africa obtained from collections of these species held in the Natural History Museum, London. Our final dataset comprised 8339 records (from 1583 locations) documenting the distribution of dung beetles in South Africa, of which 858 records (from 533 locations) document the distribution of the five species released at more than 100 sites in Australia (table 1).

### (c) Climate data

We used a global meteorological dataset that grids the world into 10′×10′ latitude–longitude grid cells (New *et al*. 2002) as the basis for constructing CEMs. The dataset records mean monthly values of a range of meteorological data, including temperature and precipitation, for each grid cell. We converted these monthly values into 16 parameters that are commonly used in climate-matching studies to characterize climate at a given location: mean annual temperature; temperature of the coolest month; temperature of the warmest month; annual temperature range; mean temperature of the coolest quarter; mean temperature of the warmest quarter; mean temperature of the wettest quarter; mean temperature of the driest quarter; annual precipitation; precipitation of the wettest month; precipitation of the driest month; coefficient of variation of monthly precipitation; precipitation of the wettest quarter; precipitation of the driest quarter; precipitation of the coolest quarter; and precipitation of the warmest quarter.

### (d) Analysis

#### (i) Predicting Australian distribution from Australian climate

We expect climate to be the major constraint on distribution in Australia given that there is no dispersal limitation (we are considering the outcome of human-mediated releases), few natural enemies (populations were screened for parasites and diseases prior to release) and no resource limitation (beetles were released at sites where cattle dung was abundant). We would therefore expect the distribution in Australia of each dung beetle species to be highly predictable from climate parameters.

For each species, we tested this prediction by constructing a CEM describing the relationship between the outcome of each release in Australia (whether a population persisted or not) and the 16 climate parameters calculated for the grid cell in which each release took place. The Australian dung beetle data are true presence/absence data: a species was absent only if it was released at a site but failed to persist. Standard approaches for modelling presence–absence data are therefore appropriate (Keating & Cherry 2004; Barry & Elith 2006; Pearce & Boyce 2006). We used boosted regression trees (BRT) to model distribution–climate relationships because of their focus on accurate prediction, suitability for exploratory analyses such as this (we had no strong *a priori* predictions about which combination of climate parameters might control each species distribution), and because they automatically allow for interactions and nonlinear relationships (De'ath 2007; Elith *et al*. 2008). BRTs have been shown to have as good, or better, predictive accuracy than other methods commonly used to construct CEMs (Elith *et al*. 2006). We used the gbm package in R (R Development Core Team 2004) to fit BRT models, allowing for up to three-way interactions, specifying a shrinkage rate (0.002) that ensured the models were fitted with more than 1000 trees (Elith *et al*. 2008), and using fivefold cross-validation to identify the optimal number of trees to minimize over-fitting.

To assess the predictive accuracy of the CEM models, we randomly divided the data for a given species into two groups, using 75 per cent of observations as training data to fit the model and the remaining 25 per cent as test data. Having fitted the model to the training data, we used that model to predict the outcomes for the test data, and then assessed the predictive accuracy of the model by calculating the area under the receiver operating curve (AUC). AUC is a measure of the likelihood that a presence will have a higher predicted value from the model than an absence. Rather than specifying a threshold for converting predicted probabilities into either presences or absences, AUC provides a measure of how well the model discriminates the presence and absence across all possible thresholds. An AUC value of 0.5 would indicate that a model has no ability to discriminate among these classes (i.e. it performs no better than chance), a value of 1 would indicate that a model always correctly assigns presences a higher probability than absences, while a value of −1 would indicate that a model always incorrectly assigns absences a higher probability than presences.

AUC values varied from one model run to the next depending on the random assignment of observations to either the training or test data, and because BRT models have a stochastic component that leads to slight variations in model output. To allow for this variation, we repeated the above process 100 times, in each case constructing a different training and test dataset at random with which to assess predictive accuracy (we were limited to 100 repeat runs because each run took a large amount of computational time). The output from these 100 runs is a distribution of AUC values that incorporates the variability associated with random sampling in order to test predictive performance on independent data, and the stochasticity associated with BRT models. We used this distribution of AUC values to summarize the degree to which the modelled climate parameters could predict dung beetle distributions in Australia.

#### (ii) Predicting South African distribution from South African climate

If climate constrains distribution in the native range, then distribution in South Africa should also be predictable from climate parameters. A key difference between the South African and Australian data, however, is that for the latter we have true presence/absence data, whereas for the former we have only locations at which the species were collected (presence-only data). One approach to constructing CEMs using presence-only data is to generate pseudo-absences by randomly picking locations where the species have not been recorded and treating these as absences in the model (Pearce & Boyce 2006). This type of sampling, however, means that standard approaches used to model true presence/absence data are not appropriate (Keating & Cherry 2004), and using these approaches may introduce an additional source of uncertainty.

Nevertheless, we have a database recording 1583 locations in South Africa where dung beetles were collected, with multiple species collected at most locations (there are 8339 species×location records). Rather then generating pseudo-absences, we used these 1583 locations to construct presence/absence records for each species on the grounds that locations where a species was not collected were at least sites visited by people with the intention of collecting dung beetles. The absence of a species at a location visited by dung beetle collectors need not imply a true absence—they may have chosen not to collect the species at that site even though it was present—but given that multiple species were collected at most sites, suggesting an element of non-selective sampling, many of the locations where a species was not collected are likely to be true absences.

We would like to proceed as if the South African dung beetle database recorded true presence/absence, which would allow us to fit BRT models comparable with those fitted to the Australian data. However, because the South African data contain an unknown number of false absences, we fitted a CEM appropriate to presence-only data and compared the predictive ability of this model with an equivalent model that assumes true presence/absence. To model presence-only data correctly, we used the Lancaster–Imbens method (eqn (18) in Keating & Cherry 2004), which is a modified form of logistic regression that accounts for false absences by estimating these from the data using an appropriate conditional probability. Keating & Cherry (2004) noted that this method solves the problem of modelling presence-only data, but recommend its use only with continuous predictor variables, which is the situation here (the model can experience convergence problems with categorical predictor variables). We used the same set of climate variables as described previously, included as main effects only, and estimated the parameters for each climate variable, along with the proportion of false absences (parameter *q* in eqn (18) of Keating & Cherry 2004), using maximum likelihood. We compared this with a standard logistic regression model, which assumes true presence/absence, using the same climate variables included as main effects. Both models were fitted to the full South African datasets, with their predictive ability compared using AUC calculated from the predicted and actual distributions. These two models differ only in their assumption about the underlying sampling distribution (presence-only versus true presence/absence), allowing us to test whether assuming true presence/absence degrades predictive performance relative to a model that properly accounts for false absences. We also compared the fit of a BRT with the same data, which assumes true presence/absence but is more flexible than logistic regression in allowing for nonlinear relationships and interactions. We show below that assuming true presence/absence does not degrade the predictive performance and that, of these three models, BRT has greater predictive ability. We therefore used BRT to model the distribution–climate relationships in South Africa, assessing their predictive accuracy using training and test datasets as we did for Australia.

#### (iii) Predicting distribution in Australia from climate in South Africa

The key test of the validity of the CEM approach is that a CEM derived in South Africa should predict distribution in Australia about as well as a CEM derived in Australia. To test this, we used the BRT models built on the training data in South Africa (as described above) to predict the distribution of test data in Australia, and assessed the predictive performance using AUC in the same way. This ensured that, for each species in Australia, our tests of predictive performance based on the models constructed in South Africa and Australia were directly comparable: we chose a random 75 per cent of the observations in each region as training data and tested the predictive accuracy of these models on the test data in Australia, repeating this process 100 times.

The use of AUC as a measure of predictive performance has been criticized, particularly in its application to species distribution modelling (Lobo *et al*. 2008). Two key criticisms are: (i) AUC avoids the issue of setting a threshold for establishing class membership, which may not be desirable. The lack of a threshold means, for example, that AUC weights omission and commission errors equally, and that predictive performance is assessed over all thresholds, some of which may not be relevant. (ii) In distribution modelling, AUC scores are sensitive to the geographic extent of the study region. Including more sites that fall increasingly outside the environmental domain of a species will lead to higher AUC scores because those absences will tend to be well predicted. As a consequence, AUC cannot be used to compare model accuracy among species because the outcomes will be sensitive to the area sampled and the degree to which species occupy that area.

AUC scores can, however, be used to compare the performance of the models constructed in Australia and South Africa in predicting Australian distributions because both models are predicting to precisely the same sets of test data. Furthermore, in using a model developed in South Africa to predict distribution in Australia, there is no criterion for establishing a threshold because the baseline probability of species occurrence in South Africa is unknown: the South African data are presence-only, which means the baseline probability is arbitrarily set by the number of pseudo-absences (Pearce & Boyce 2006). In this case, scores that assess performance across all thresholds, such as AUC, provide a sensible measure.

As an additional measure of model performance, we calculated the Hosmer–Lemeshow goodness-of-fit statistic (Hosmer & Lemeshow 1989) for models fitted to the full set of distributional data for each species. The Hosmer–Lemeshow statistic is chi-squared distributed with a significant value indicating a lack of fit arising from a poorly specified model.

## 3. Results

**Figure 2:**
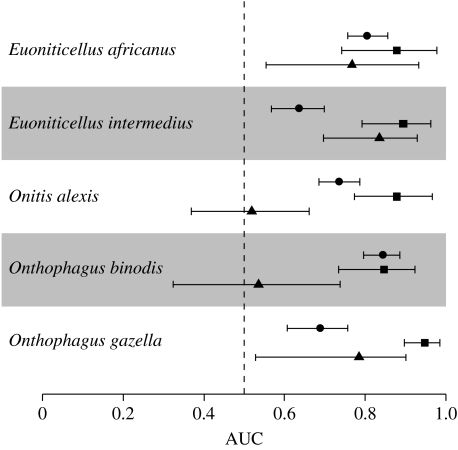
Comparison of the predictive accuracy (using area under the receiver operating curve, AUC) of CEMs for five species of dung beetle released in Australia. The mean AUC and 95% quantiles from 100 runs are shown (see text). Circles show the mean predictive accuracy of models derived in South Africa for predicting South African distributions, squares show the mean predictive accuracy of models derived in Australia for predicting Australian distributions and triangles show the mean predictive accuracy of models derived in South Africa for predicting Australian distributions.

The distribution of successful dung beetle releases in Australia was highly predictable from Australian CEMs constructed using BRT (figure 2), with predictive performance, as measured by AUC applied to independent data, much closer to 1 than 0.5 for all model runs across all species. Similarly, with the exception of one species, Hosmer–Lemeshow statistics showed no evidence of a lack of model fit (table 3). The lack of fit for *O. alexis* implies a poorly specified model, which could be due to nonlinear relationships or interactions unaccounted for in the model, or the absence of important explanatory variables. BRTs account for nonlinear relationships and we included up to three-way interactions in the model, so the most likely reason for the lack of fit is that the distribution of *O. alexis* in Australia is influenced by other non-climatic variables. One factor that could be important is variation in the number of individuals released, so we included the (log-transformed) number of beetles released at each site as an additional variable in the *O. alexis* model. This updated model showed no evidence of a lack of fit (Hosmer–Lemeshow statistic=14.0, *p*=0.08) implying that, for this species, the variation in release effort, in addition to climate variables, determined the distribution at Australian release sites.

The estimated number of false absences in the South African data ranged from 6 to 23 per cent (table 2), but ignoring these and treating the data as true presence/absence did not degrade predictive performance. Indeed, predictive performance was slightly better for two species (and no worse for the other three species) using logistic regression compared with the Lancaster–Imbens method. BRT models had greater predictive performance for all species, suggesting that while there was no cost to treating the data as true presence/absence, there were important nonlinear relationships and/or interactions that were captured using the more flexible modelling approach. South African CEMs did better than chance at predicting dung beetle distributions in South Africa for all species (figure 2), but Hosmer–Lemeshow statistics revealed a significant lack of fit for all models (table 3). This is most likely because climate parameters alone are insufficient to model South African distributions adequately, with key non-climatic variables missing from the models.

The predictive accuracy of South African CEMs was in all cases lower than Australian CEMs for predicting distribution in Australia, although for two species (*E. intermedius* and *E. africanus*) there was considerable overlap in the AUC scores (figure 2). For these species, South African CEMs do an almost equally good job of predicting distribution in Australia as Australian CEMs. For a third species, *O. gazella*, the South African CEM predicted distribution in Australia better than chance, but with significantly lower predictive accuracy than the Australian CEM. For the remaining two species (*O. binodis* and *O. alexis*), the South African CEMs performed significantly worse than the Australian CEMs, and were not significantly better than chance in predicting distribution in Australia. Hosmer–Lemeshow statistics also revealed a highly significant lack of fit when South African CEMs were used to model Australian distributions (table 3).

## 4. Discussion

The use of CEMs to forecast range shifts in response to climate change rests on several assumptions, including the fundamental one that climate limits species distributions (Parmesan *et al*. 2000). Dung beetle introductions to Australia are unique in that they involved the purposeful removal of key non-climatic constraints (dispersal limitation, natural enemies and resource limitation), such that we expect climate to be the major factor limiting distribution at Australian release locations. This is supported by our results: CEMs constructed using training data in Australia and used to predict Australian distributions in independent test data had very high predictive accuracy for all five species (figure 2), and models constructed using climate parameters alone fitted the data well (with the exception of *O. alexis* discussed above; table 3). For most species, the locations where they failed to persist were geographically distinct from those where they persisted, with failures more common at higher latitude coastal sites, which correspond to generally cooler, wetter parts of the country (figure 1).

These Australian results set the standard for how we expect CEMs to perform when climate actually does limit distribution. If climate also limits distribution in the native range, then we expect a CEM constructed in South Africa to predict distribution in Australia about as well as the Australian CEMs. This was the case for only two of the five species (figure 2); for the remaining three species, the predictive performance of models developed in South Africa was significantly worse than those developed in Australia, and for two species the South African models performed no better than chance.

Models in the two regions were constructed using the same methods, and we have shown that differences in predictive performance are unlikely to be due to false absences in the South African data. Other deficiencies in the data could still contribute to the observed differences. In particular, three of the dung beetle species we studied (*E. intermedius*, *O. alexis* and *O. gazella*) have native distributions that extend beyond South Africa. In addition to the 604 location records for these species in South Africa (table 1), we collated a further 123 records from outside South Africa. This means that the distributional data used to model these species could be a biased subset that excludes climate zones found outside South Africa. We chose not to use these additional records primarily because locations within South Africa were well sampled in the dung beetle database, while the records outside South Africa were derived from museum specimens that were widely geographically scattered. We treated locations visited by a collector in South Africa where a species was not recorded as an absence. Using data from a wider geographic area would necessitate generating random pseudo-absences, which, given the scattered distribution of the additional records, would almost certainly lead to a higher proportion of false absences and potentially greater uncertainty. Moreover, the climate over much of Australia and South Africa is comparable, which is why South Africa was chosen as the source for dung beetle introductions with species selected on the basis of their climatic compatibility (Bornemissza 1976). Indeed, there is little indication in our results (figure 2, table 3) that species whose ranges extend beyond South Africa (*E. intermedius*, *O. alexis* and *O. gazella*) were modelled any worse than those confined to South Africa (*E. africanus* and *O. binodis*).

While other biases in the distribution records could affect model performance (Barry & Elith 2006), our results suggest a key reason why South African CEMs for three species transferred poorly to Australia is that different factors limit distribution in the two regions. Indeed, while most Australian CEMs provided a good fit to the Australian data, as expected if climate limits distribution, the South African CEMs all showed a significant lack of fit, implying poor model specification most likely due to the absence of key non-climatic variables that limit distribution in the native range. While the composition of the dung beetle assemblages across South Africa is linked to climatic gradients, other edaphic factors, such as soil conditions, and barriers to dispersal associated with the geological and climatic history of the region appear to play a key role (Davis *et al*. 2008). This absence of non-climatic variables in the South African models significantly affected the transferability of CEMs in three of the five species.

Considerable effort has gone into identifying and understanding the major sources of uncertainty associated with the use of CEMs to project current and future species distributions (Guisan & Thuiller 2005; Araújo & Guisan 2006; Heikkinen *et al*. 2006; Botkin *et al*. 2007; Beale *et al*. 2008). At its core, however, is the assumption that climate limits distribution, and perhaps the greatest uncertainty surrounds whether this assumption holds for the species we model. Our results suggest that climate may not be the major factor limiting native distributions in some, if not most, cases. Indeed, from our small sample we would conclude that models based on climate parameters alone may have some value in forecasting species distributions under altered climates in 40 per cent of cases, and have uncertain value, or do little better than chance in the remaining 60 per cent. Moreover, it may be difficult to identify *a priori* which species will, or will not, meet the required assumptions. In our study, species in the same genus, which might be expected to share traits in common and to respond in a similar manner relative to species in different genera, showed some tendency to do so (figure 2), although we do not know why.

While many studies use models based on climate parameters alone to predict distributions, the inclusion of other environmental, biotic or dispersal variables in these models can improve fit and increase predictive accuracy (Heikkinen *et al*. 2006). Explicitly incorporating non-climatic factors in species distribution models acknowledges that climate may not, or may only partly, limit distribution. By modelling these non-climatic factors, the aim is to remove their effects statistically and to uncover the underlying true climate–distribution relationships, which can then be used in forecasting. Given that factors other than climate limit distribution for at least some species, inclusion of non-climatic variables in species distribution models is critical to uncovering the underlying climate–distribution relationships. Climate may limit distribution in some species, but unless we can identify these *a priori*, the only alternative is to ensure that all models account for relevant non-climatic factors. Identifying these factors, quantifying them in a meaningful way and dealing with likely interactions between non-climatic and climatic variables remains a challenging task (Heikkinen *et al*. 2006).

## Figures and Tables

**Table 1:** Sample sizes for the number of locations where dung beetle species were released and either persisted or failed to persist in Australia, and the number of presence-only locations from which distribution models in the native range (South Africa) were derived.

species	Australia		South Africa
	
	no. of release locations where species persisted	no. of release locations where species failed to persist	no. of presence locations
*Euoniticellus africanus*	69	45	138
*Euoniticellus intermedius*	1047	57	192
*Onitis alexis*	221	61	294
*Onthophagus binodis*	93	48	116
*Onthophagus gazella*	981	73	118

**Table 2:** The proportion of false absences in the South African data, estimated using the Lancaster–Imbens method (see text), and AUC scores assessing the predictive performance of CEMs fitted to the South African data using the Lancaster–Imbens method, logistic regression or BRT.

species	proportion of false absences	Lancaster–Imbens	logistic	BRT
*Euoniticellus africanus*	0.23	0.81	0.82	0.90
*Euoniticellus intermedius*	0.12	0.68	0.68	0.79
*Onitis alexis*	0.16	0.71	0.71	0.83
*Onthophagus binodis*	0.21	0.84	0.85	0.92
*Onthophagus gazella*	0.06	0.73	0.73	0.80

**Table 3:** Hosmer–Lemeshow goodness-of-fit statistics for CEM models constructed in Australia and South Africa, and predicting dung beetle distributions in these regions. (The statistics are chi-squared distributed with eight degrees of freedom, for which the critical value determining significance at the 0.05 level is 15.5. Values greater than this indicate models with a significant lack of fit, with significance indicated as ^**^p<0.01, ^***^p<0.001.)

species	Australian CEM predicting Australian distribution	South African CEM predicting South African distribution	South African CEM predicting Australian distribution
*Euoniticellus africanus*	12.1	23.9^***^	82.3^***^
*Euoniticellus intermedius*	8.2	60.1^***^	4397.7^***^
*Onitis alexis*	17.5^**^	48.4^***^	1179.2^***^
*Onthophagus binodis*	14.8	22.1^**^	939.8^***^
*Onthophagus gazella*	8.3	28.0^***^	5415.2^***^
